# The prognostic values of the expression of Vimentin, TP53, and Podoplanin in patients with cervical cancer

**DOI:** 10.1186/s12935-017-0450-6

**Published:** 2017-09-07

**Authors:** Jiaying Lin, Jiaqi Lu, Chao Wang, Xiaohong Xue

**Affiliations:** 1 Department of Assisted Reproduction, Shanghai Ninth People’s Hospital, Shanghai Jiao Tong University, School of Medicine, 639 Zhizaoju Road, Shanghai, 200011 China; 2 Department of Gynecology, Obstetrics and Gynecology Hospital, Fudan University, 419 Fangxie Road, Shanghai, 200011 China; 3 Department of Pathology, Obstetrics and Gynecology Hospital, Fudan University, 419 Fangxie Road, Shanghai, 200011 China

**Keywords:** Vimentin, EMT, TP53, Podoplanin, Prognostic marker, Cervical cancer

## Abstract

**Purpose:**

Epithelial–mesenchymal transition (EMT), TP53, and Podoplanin have been implicated in the tumorigenesis and metastasis of human cancers. Nevertheless, the clinical significance of these markers in cancer patients is still not clear. In this study, we sought to determine the prognostic values of Vimentin, TP53, and Podoplanin in patients with cervical cancer.

**Methods:**

Quantitative real-time polymerase chain reaction (qRT-PCR) and Western blot analysis were performed to determine the messenger RNA and protein expression levels of Vimentin, TP53, and Podoplanin, respectively, in cervical squamous cell carcinoma and adjacent normal cervical tissues. Additionally, the expression levels of Podoplanin were also measured in 130 cervical cancer patients (FIGO stages Ib1–IIa2) using immunohistochemistry (IHC) staining.

**Results:**

The mRNA expression levels of Vimentin, TP53, and Podoplanin were considerably elevated in cervical cancer tissues, compared with those in the adjacent normal cervical tissues. Additionally, the protein expression levels of Vimentin were closely correlated with the age of onset (P = 0.007), lymph node metastasis (P = 0.007), lymphatic invasion (P = 0.024), disease recurrence (P < 0.001), and the clinical prognosis of patients with cervical cancer (P < 0.001). Our multivariate analysis also suggests that Vimentin is an independent marker for survival in cervical cancer patients. Furthermore, the expression levels of Vimentin are negatively correlated with the proliferation marker Ki67 expression.

**Conclusions:**

Our data show that Vimentin can serve as an independent prognostic marker for cervical cancer patients with primary surgery.

*Registration number* ChiCTR-TRC-06000236 Registered 15 December 2006

## Background

With approximately 470,000 new cases and 233,000 deaths each year, cervical cancer is one of the most common cancers found in women globally [1]. 85% of these cases and deaths occurred in developing countries, including China [2]. The malignant transformation of human cells is characterised by their capacity to invade neighbouring tissues and metastasize to distant organs. Cancer invasion and metastasis are associated with a physiological process, epithelial–mesenchymal transition (EMT), by which epithelial cells lose their cell polarity and cell–cell adhesion, and gain a mesenchymal phenotype [3]. EMT plays a considerable role in normal embryonic development as well as wound healing. It allows epithelial cells to undergo dramatic morphological and biochemical changes, which results in the loss of cell–cell and cell–extracellular matrix connections and enables the cells to migrate to other organs in the body throughout the physiological process of tissue maintenance and repair [4]. Notably, EMT plays a fundamental role in the disease progression including the acquisition of the invasive and metastatic potentials for not only head and neck squamous cancer cells but also breast and hepatic cancer cells [3, 5–7]. The loss of epithelial phenotype and gaining of mesenchymal properties enable cancer cells to spread to distant organs of the body at a much faster pace [8, 9]. Vimentin is a key protein that composes the interstitial cell skeleton. Its expression level is upregulated during EMT and closely correlated with cancer invasion and metastasis [7, 10]. Additionally, Vimentin has also been involved in cell cycle regulation and adhesion [11], which further validates its role in the development and progression of human cancers.

Our previous studies have shown that cervical cancer stem-like cells, which are extremely resistant to chemoradiotherapy, play a crucial role in contributing to the mechanism of metastasis in human cervical cancer. We demonstrated that cervical cancer stem-like cells exhibit typical EMT features, including upregulated expression of EMT-related genes such as Vimentin, Twist 1, Twist 2, Snail 1 and Snail 2 and the potential to migrate through the basal membrane [12]. Intriguingly, Vimentin has also been reported to form a complex with TP53 in the cytoplasm and then suppress the translocation of TP53 into the nucleus, thereby inhibiting the function of TP53 in promoting apoptosis [13]. These findings suggest that TP53 also plays an important role in the EMT pathway.

TP53 is a tumour suppressor nuclear protein that exerts its anti-cancer function by inhibiting cell cycle progression [14]. Compared with wild-type TP53, mutant TP53 exhibits a longer half-life and is localized in the nucleus, allowing us to readily examine its expression using immunohistochemistry (IHC). Consistently, previous reports have suggested a strong correlation between TP53 mutations and immunohistochemical detection of TP53 nuclear reactivity in human cancer tissues. TP53 mutations are undoubtedly involved in the development and progression of human cancers. However, the clinical prognostic values of TP53 mutations in cervical cancer remains controversial [15]. It has been suggested that TP53 overexpression is correlated with an unfavourable prognosis in cervical cancer patients [16, 17]. Nevertheless, other groups have also reported that TP53 mutations do not display important prognostic values in the management of cervical cancer patients [18, 19].

Invasion and migration of cancer cells into the nearby tissues as well as their ingress into the microvasculature of the lymphatic system are crucial steps for the lymphatic dissemination of malignant tumours [20]. Podoplanin, a small membrane mucin-like type I glycoprotein, is well expressed in lymphatic endothelial cells, but not in blood vessel endothelium. Thus, it has been widely used as a specific marker for lymphatic endothelial cells and lymphangiogenesis in various species [21]. Podoplanin forms a complex with ezrin/moesin of the Ezrin, Radixin and Moesin protein family through the interaction of its cytoplasmic domain with the cytoskeleton protein, actin. This interaction enables Podoplanin to activate GTPase RhoA and its related RhoA-linked kinase, thereby promoting EMT [22]. However, Wicki et al. have also reported that Podoplanin can activate the rearrangement of the cytoskeleton protein, actin, thereby promoting tumour cell migration in the absence of the involvement of EMT [23].

In this study, we aimed to investigate the clinicopathologic properties of the expression of Vimentin, TP53 and Podoplanin in human cervical cancer tissues. Our findings will reveal the prognostic values of these protein markers in cervical cancer patients and may provide important guidelines for the management of the patients in the future.

## Materials and methods

### Clinical samples

130 cervical cancer tissues collected in the Obstetrics and Gynecology Hospital of Fudan University (Shanghai, China) between November 2007 and December 2012 were analysed to determine the clinical staging and clinicopathological characteristics of the cervical cancers. All protocols were carried out in accordance with the altered International Federation of Gynecology and Obstetrics staging system (FIGO) regarding cervical cancer published in 2009. All patients enrolled in this study were diagnosed with only gynaecological tumour(s). Additionally, they did not received preoperative radiotherapy, chemotherapy, or hormonal therapy. Following the surgery, 93 patients underwent adjuvant radiotherapy and (or) chemotherapy. All participants of this study provided their written informed consent regarding the use of the clinical materials for the research we described here. Additionally, this study was approved by the Research Ethics Committee of Fudan University, Shanghai, China. In accordance with the ethical and legal standards, all of the samples were rendered anonymous by removing all identifiers. The patients’ characteristics related to the samples have been illustrated in Table 1. The follow-up time in respect of the initial cervical cancer group fell between the ranges from 18 to 89 months, whereas the median follow-up time was 53.2 months.

**Table 1:** Primer sequences used for RT-PCR in this study

Name	Forward primer sequence	Reverse primer sequence
Vimentin	CCAGGCAAAGCAGGAGTC	GGGTATCAACCAGAGGGAGT
TP53	ACCCAGGTCCAGATGAAG	CACTCGGATAAGATGCTGA
Podoplanin	CCAGGAGAGCAACAACTCAA	TCCTCATGTTTGTGCAGGAG
GAPDH	ATCATCCCTGCCTCTACTGG	CCCTCCGACGCCTGCTTCAC

### Quantitative real-time-polymerase chain reaction (qRT-PCR)

Total RNA was purified from cervical cancer tissues as well as from surrounding normal cervical tissues using the Trizol reagent (Invitrogen, Carlsbad, CA, USA) according to the manufacturer’s protocol. The concentration of the purified RNA was measured with a NanoDrop ND-1000 spectrophotometer (Thermo Scientific, Wilmington, DE, USA). Following the first-strand cDNA synthesis with the Reverse Transcription Kit (Invitrogen), qRT-PCR was performed using the SYBR real-time PCR Kit (Invitrogen) according to the manufacturer’s protocol. The primers used in the PCR amplification were listed in Table 1.

### Immunohistochemistry

Formalin-fixed, paraffin-embedded tissues were sectioned to a thickness of 4 μm, mounted onto glass slides, deparaffinized with xylene and then rehydrated through the graded ethanol series (100, 95 and 70%) to deionized H2O. The expression of Vimentin, TP53, and Podoplanin was determined with IHC staining. The antibodies used in the IHC staining included mouse monoclonal antibodies against Vimentin (dilution 1: 200; Dako, Denmark), TP53 rabbit monoclonal antibody (1:100 dilution, Carpinteria, CA), and mouse monoclonal antibody against Podoplanin (1:100 dilution, Dako). Briefly, retrieval of antigens was done through steam heat for 20 min in a 0.01 M trisodium citrate buffer (pH 6.0). The slides were then immersed in the ChemMate peroxidase-blocking solution (Dako) for 10 min at room temperature to block endogenous peroxidase activity. The samples were subjected to immunostaining with primary antibodies for 2 h, followed by incubation with HRP-labeled anti-mouse or anti-rabbit secondary antibodies. Visualization of immunoreactive proteins was carried out using 3,3-diaminobenzidine (Sigma-Aldrich) as chromogen and nuclei were counterstained with Mayer’s hematoxylin. Following dehydration through an ethanol series (70, 90 and 100%), the slides were mounted and evaluated by two pathologists (S.L. and C.W.) independently as described below. To assess the quality control of our IHC staining protocol, normal cervical tissue staining and an isotype control (Dako) were used as positive and negative controls, respectively.

Two pathologists (S.L. and C.W.) who were unaware of the clinical data as well as other immunohistochemical findings carried out semiquantitative evaluation of the slides. When there were differences in scoring, a consensus was reached through discussion between the two pathologists. The expression levels and subcellular localization of Vimentin, TP53, and Podoplanin were determined using the positive and negative controls as a reference. At least 1000 cells in five randomly chosen areas of the tumour tissues were analysed in each section at a ×400 magnification to obtain the labelling indices (percentage of positive cells).

Vimentin expression was scored as positive when cytoplasmic or nuclear staining of the cells was observed in greater than 10% of the tumor tissues [24]. Tumor tissues with a positive immunohistological staining of at least 50% of tumor cells were defined to have positive TP53 expression [18, 25]. Podoplanin expression was considered positive when there was moderate or strong immunoreaction in more than 10% of the cells [26].

### Statistical analysis

Statistical analysis was carried out using SPSS version 19.0 software (SPSS Inc., Chicago, Ill., USA. Disease-free survival was defined as the interval falling between the date of surgery and the date of tumor recurrence or the date of the most recent follow-up with no proof of tumor recurrence. At the time of the previous visit for regular follow-ups, a censor was performed on overall survival time. Chi squared test or Fisher’s exact test was performed to compare the difference between groups. Cox’s proportional hazards model was built to calculate the risk ratios and their corresponding 95% confidence intervals (CIs). The associations of the gene expression with disease-free and overall survival was evaluated based on the Univariate and multivariate Cox’s proportional hazards models. Survival rates were estimated using the Kaplan–Meier method and log-rank test. The results were considered statistically significant, when P values are less than 0.05.

## Results

### Patient demographics

In order to evaluate the expression of Vimentin, TP53, and Podoplanin, 130 samples from cervical cancer patients were used in this study. The clinicopathological characteristics of the patients are presented in Table 1. The patients have a median age at 48 years old, ranging from 28 to 70 years. According to the 2009 FIGO criteria, the patients were diagnosed with cervical cancers of different clinical stages: stage Ib1 (57.7%), stage Ib2 (10.7%), stage IIa1 (23.1%), and stage IIa2 (8.5%). Additionally, the patients had cervical cancers of different degrees of differentiation: well differentiated (11 cases), moderately differentiated (97 cases), and poorly differentiated (22 cases). A total of 22 patients had lymph node metastases, while the other 108 patients did not.

### The mRNA expression of Vimentin, TP53, and Podoplanin in cervical cancer

**Fig. 1:**
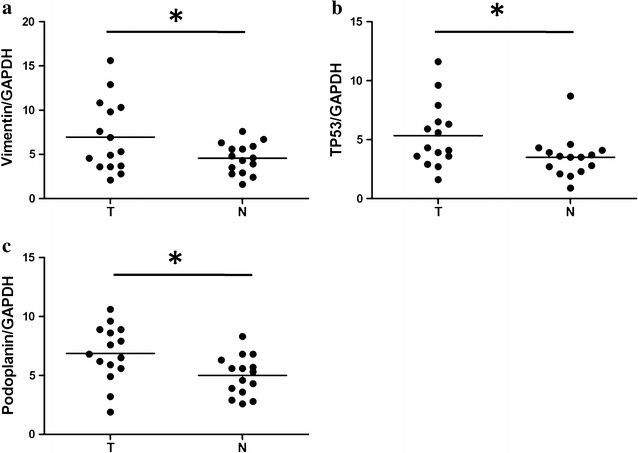
Vimentin (**a**), TP53 (**b**), and Podoplanin (**c**) expression levels in cervical cancer tissues and adjacent non-tumorous tissues

The mRNA expression levels of Vimentin, TP53, and Podoplanin were examined using qRT-PCR in cervical cancer tissues and surrounding normal cervical tissues from fifteen randomly selected cases of cervical cancer patients. Our results demonstrated that cervical cancer tissues exhibit considerably higher mRNA expression levels of Vimentin, TP53, and Podoplanin than the normal tissues (P < 0.05; Fig. 1).

### The expression of Vimentin, TP53 and Podoplanin in cervical cancers

**Fig. 2:**
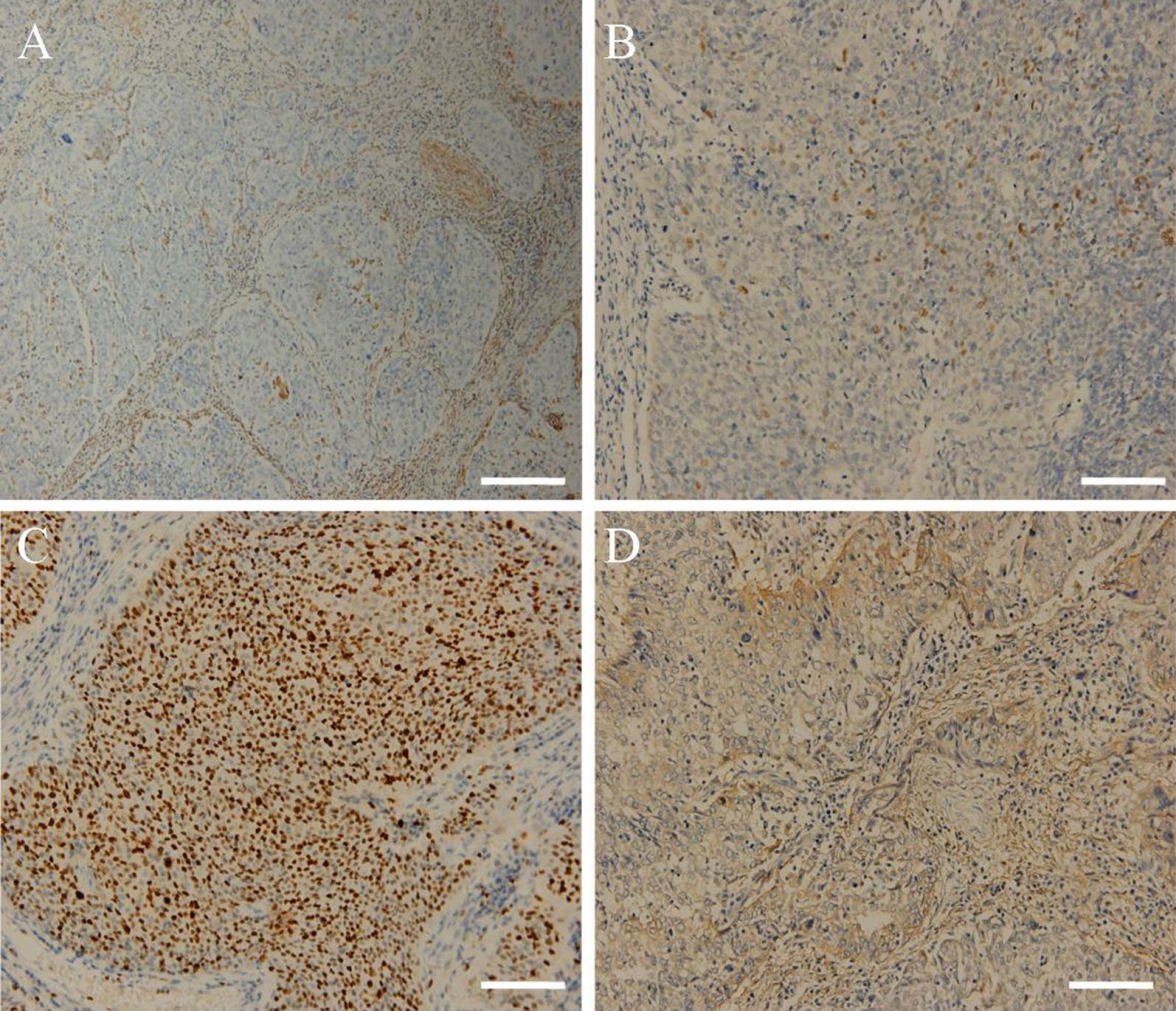
Exemplary photographs of Vimentin (**A**), tumor protein P53 (TP53) (**B**), Ki67 (**C**) and Podoplanin (**D**) immunohistochemical staining in primary tumors central area (×200)

In order to examine the expression of Vimentin, TP53 and Podoplanin in cervical cancer, IHC analysis was performed as described in the “Materials and methods”. Representative images defined as positive staining of the three proteins were shown in Fig. 2. Cells with positive Vimentin expression display yellow or brown granules in the cytoplasm that are close to the membrane. The presence of yellow or brown granules in the nucleus and cytoplasm of cancer cells is indicative of positive TP53 expression. On the other hand, Podoplanin is expressed primarily in the cytoplasm as well as on the plasma membrane of the tumour cells (Fig. 2).

### The association of the expression of Vimentin, TP53 and Podoplanin with the clinicopathological characteristics of cervical cancer

We next investigated whether the expression of Vimentin, TP53, and Podoplanin is correlated with several clinicopathological features in cervical cancer. Our data showed that Vimentin expression is considerably associated with the onset age (P = 0.007), lymph node metastasis (P = 0.007), lymphatic invasion (P = 0.024), disease recurrence (P < 0.001), and the clinical prognosis of patients (P < 0.001) (Table 2).

**Table 2:** Correlation of immunohistochemical study of Vimentin, TP53 and Podoplanin with clinicopathological factors in cervical cancer

Characteristics	Total (n = 130)	Vimentin (n = 130)		Chi square test P value	Fisher’s exact test P value	P53 (n = 130)		Chi square test P value	Fisher’s exact test P value	Podoplanin (n = 130)		Chi square test P value	Fisher’s exact test P value
		Positive (n = 35)	Negative (n = 95)			Positive (n = 26)	Negative (n = 104)			Positive (n = 61)	Negative (n = 69)		
Age at diagnosis (years)													
<50	77	14	63	0.007	0.009	12	65	0.129	0.098	21	56	<0.0001	<0.0001
≥50	53	21	32			14	39			40	13		
Tumor size (cm)													
<4	100	24	76	0.17	0.24	16	84	0.037	0.038	44	56	0.223	0.297
≥4	30	11	19			10	20			17	13		
Histological grades													
Well/moderate	108	27	81	0.273	0.297	24	84	0.16	0.243	48	60	0.21	0.246
Poor	22	8	14			2	20			13	9		
FIGO stage													
Ia1–Ib2	89	22	67	0.404	0.265	16	73	0.396	0.266	38	51	0.155	0.187
IIa1–IIa2	41	13	28			10	31			23	18		
Lymph node metastasis													
No	108	24	84	0.007	0.015	21	87	0.726	0.771	46	62	0.028	0.035
Yes	22	11	11			5	17			15	7		
Vascular space involvement													
No	73	18	55	0.51	0.554	6	67	<0.0001	<0.0001	13	60	<0.0001	<0.0001
Yes	57	17	40			20	37			48	9		
Tumor histology													
Squamous	95	25	70	0.416	0.486	23	72	0.108	0.126	46	49	0.165	0.171
AC/ASC	29	7	22			3	26			12	17		
Others	6	3	3			0	6			3	3		
Lymphatic invasion													
Yes	57	21	36	0.024	0.029	10	47	0.536	0.347	52	5	<0.0001	<0.0001
No	73	14	59			16	57			9	64		
Squamous cell carcinoma antigen, ng/ml													
≤5	88	23	65	0.77	0.834	16	72	0.453	0.487	54	34	0.841	0.85
>5	42	12	30			10	32			25	17		
Deep stromal invasion													
Yes	69	21	48	0.337	0.429	17	52	0.16	0.191	40	29	0.007	0.009
No	61	14	47			9	52			21	40		
Positive parametrium													
Yes	10	2	8	0.607	0.464	3	7	0.411	0.418	8	2	0.029	0.045
No	120	33	87			23	97			53	67		
Positive surgical margin													
Yes	3	1	2	0.8	0.613	1	2	0.559	0.491	3	0	0.247	0.338
No	127	34	93			25	102			58	69		
Vaginal involvement													
Yes	32	8	24	0.778	0.486	8	24	0.415	0.449	18	14	0.223	0.308
No	98	27	71			18	80			43	55		
Recurrence													
Yes	15	12	3	<0.001	<0.001	7	8	0.006	0.012	12	3	0.006	0.011
No	115	23	92			19	96			49	66		
Vital status (at follow-up)													
Alive	120	27	93	<0.001	<0.001	20	100	0.001	0.004	52	68	0.004	0.006
Dead	10	8	2			6	4			9	1		
Ki67 (%) (mean ± SD)	53.3 ± 30.5	49.7 ± 31.2	62.4 ± 26.3	0.037		50.9 ± 32.1	61.2 ± 21.9	0.059		32.6 ± 34.1	41.1 ± 36.1	0.171	

*AC* adenocarcinoma, *ASC* adenosquamous carcinoma, others including undifferentiated sarcoma, neuroendocrine and glassy cell cervical carcinoma

Similarly, a significant association was observed between the expression of TP53, and tumour size (P = 0.037), vascular space involvement (P < 0.0001), disease recurrence (P = 0.006) and the clinical prognosis of cervical cancer patients (P = 0.001) (Table 2).

Moreover, Positive staining of Podoplanin was also significantly correlated with onset age (P < 0.0001), lymph node metastasis (P = 0.028), vascular space involvement (P < 0.0001), lymphatic invasion (P < 0.0001), deep stromal invasion (P = 0.007), positive parametrium (P = 0.029), disease recurrence (P = 0.006), and the clinical prognosis of patients (P = 0.004) (Table 2).

### The association of the expression of Vimentin, TP53 and Podoplanin with the proliferation of cervical cancer

In order to investigate whether there is a link between the expression of these three protein biomarkers and the proliferation of cervical cancer, we next examined the correlation of the expression of Vimentin, TP53 or Podoplanin with Ki67, a cellular marker for proliferation (Table 2). We found that Vimentin expression is closely correlated with Ki67 expression in cervical cancer tissues (P = 0.037). Nevertheless, there is no significant association between the other two protein markers and Ki67 (P > 0.05).

### Expression of Vimentin, TP53 and Podoplanin as prognostic factors in patients with cervical cancer

**Fig. 3:**
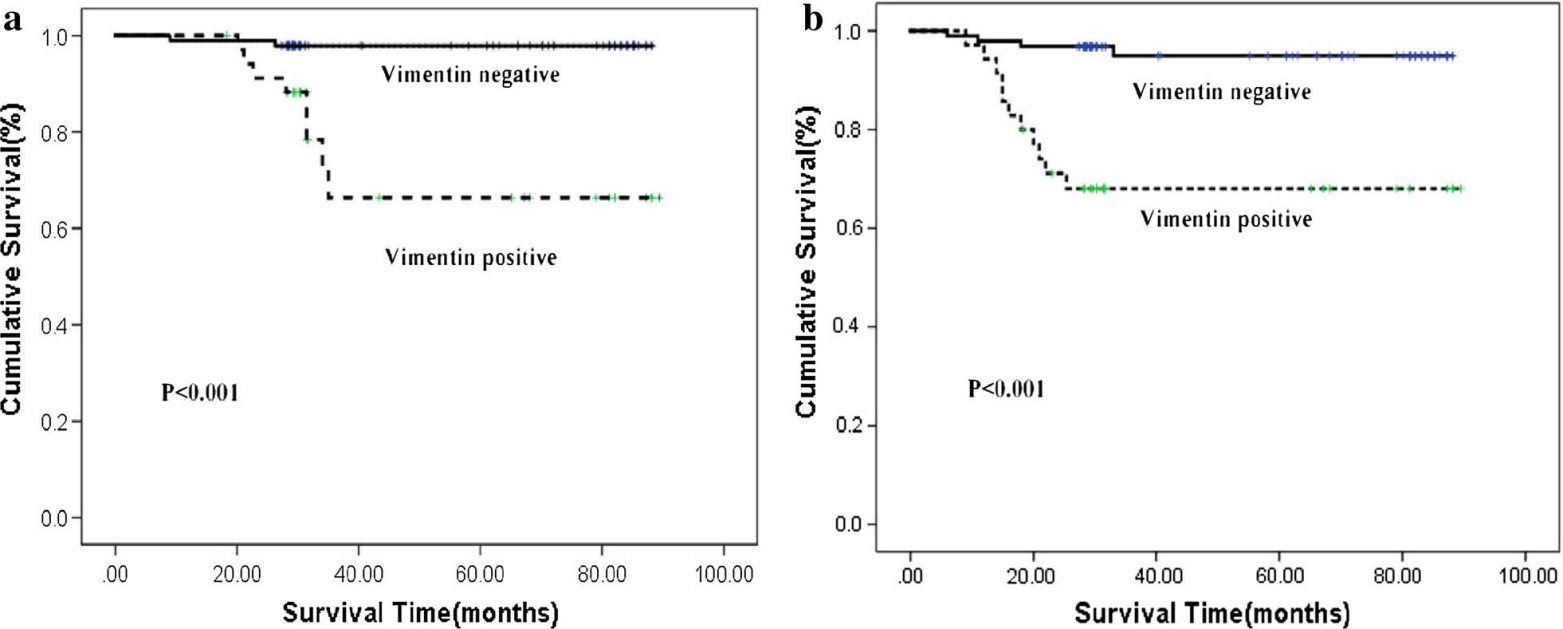
Kaplan–Meier analysis of overall (**a**) and disease-free (**b**) survival according to Vimentin expression

The cumulative OS and DFS rate of the 130 patients with cervical cancer were 92.3 and 88.5%, respectively. To evaluate the prognostic value of Vimentin, TP53 and Podoplanin in cervical cancer, we then examined the correlation between the expression of Vimentin, TP53 and Podoplanin and patients’ survival using the Kaplan–Meier estimate and log-rank test. Our data showed that patients with positive expression of Vimentin exhibit shorter OS as compared with those with negative expression (77.1% vs. 97.9%, P < 0.001). Similarly, Vimentin-positive patients display significantly shorter DFS (65.7%), compared with Vimentin-negative patients (96.8%) (P < 0.001) (Fig. 3a, b).

**Fig. 4:**
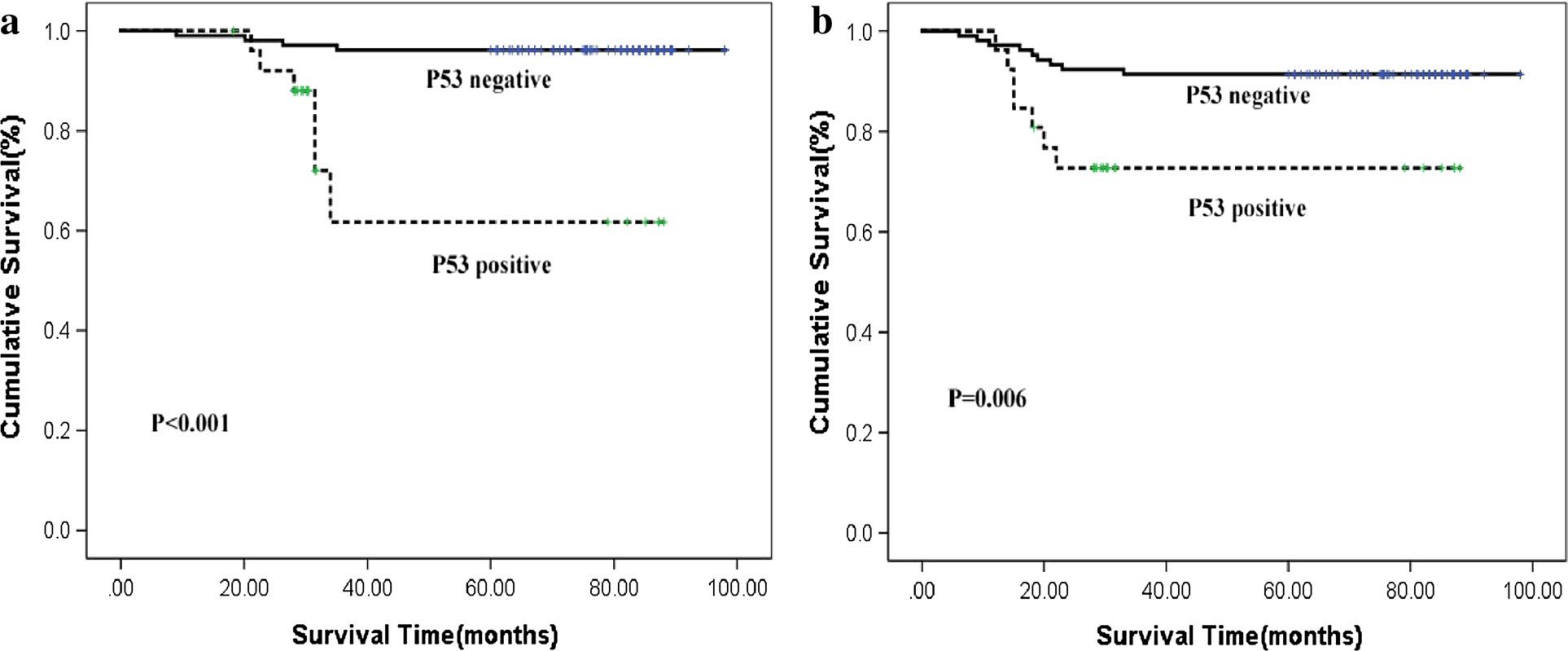
Kaplan–Meier analysis of overall (**a**) and disease-free (**b**) survival in presence or absence of TP53 expression

Additionally, we also explored the impact of TP53 expression on the OS and DFS of cervical cancer patients. The cumulative OS rate for the cervical cancer patients with positive TP53 expression (76.9%) is significantly lower than that for patients with negative TP53 expression (96.2%, P < 0.001). Similarly, cervical cancer patients with positive TP53 expression also display smaller cumulative DFS rate (73.1%), compared with those with negative TP53 expression patients (92.3%, P = 0.006) (Fig. 4a, b).

**Fig. 5:**
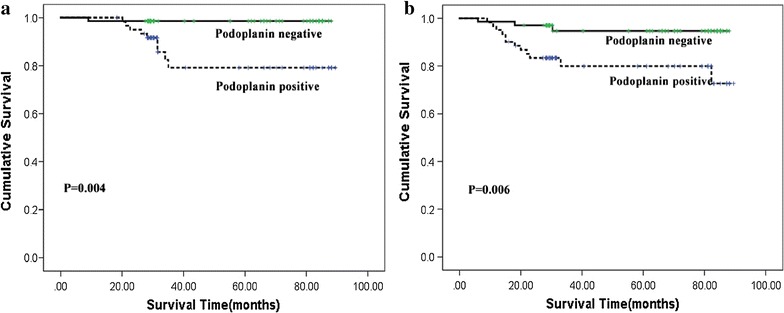
Kaplan–Meier analysis of overall (**a**) and disease-free (**b**) survival in presence or absence of Podoplanin expression

Our data also show that Podoplanin expression is negatively correlated with the OS (P = 0.004) and DFS (P = 0.006) of cervical cancer patients (Fig. 5a, b). The OS (85.2%) and DFS (80.3%) rates in the cervical cancer patients with positive Podoplanin expression are smaller than those for the patients without Podoplanin expression (OS 98.6%, DFS 95.7%).

We next employed Cox proportional hazards model to examine the clinicopathologic features of the expression of Vimentin, TP53 and Podoplanin in cervical cancer patients (Table 3). The results from our univariate and multivariate analysis suggest that age and Vimentin expression exhibit a considerable impact on the OS and DFS of cervical cancer patients (Table 3). These data indicate that age and Vimentin expression are independent prognostic factors for cervical cancer patients.

**Table 3:** Cox regression analysis for factors possibly influencing OS and DFS in patients with cervical cancer

Factors	Recurrence-free survival				Overall survival			
	Univariate		Multivariate		Univariate		Multivariate	
	HR (95% CI)	P value	HR (95% CI)	P value	HR (95% CI)	P value	HR (95% CI)	P value
Age (≥50 vs <50)	0.138 (0.029–0.648)	0.012	0.104 (0.02–0.567)	0.01	0.111 (0.023–0.527)	0.005	0.6 (0.397–0.906)	0.015
Tumor size (≥4 vs <4 cm)	1.117 (1.03–1.452)	0.002	0.156 (0.029–1.217)	0.079	1.95 (1.024-1.375)	0.001	0.336 (0.186–1.609)	0.082
Histology (well and moderate vs poor)	0.346 (0.1–1.196)	0.094	0.365 (0.086–1.543)	0.173	1.042 (0.682–1.590)	0.196	0.447 (0.128–1.563)	0.207
LN metastasis (absent vs present)	5.378 (1.556–18.58)	0.008	0.467 (0.078–2.774)	0.404	5.111 (1.479–17.659)	0.01	0.592 (0.311–1.126)	0.455
Vascular space involvement (absent vs present)	12.262 (1.553–96.82)	0.017	1.612 (0.057–38.489)	0.805	11.737 (1.487–92.552)	0.019	0.777 (0.401–1.505)	0.565
Lymphatic invasion (absent vs present)	10.125 (1.213–85.32)	0.021	7.531 (0.325–156.960)	0.239	11.894 (1.507–93.909)	0.017	1.202 (0.642–2.252)	0.746
Deep stromal invasion (absent vs present)	8.498 (1.076–67.086)	0.042	2.498 (0.323–26.631)	0.45	8.97 (1.135–70.892)	0.038	5.733 (0.593–55.392)	0.702
Podoplanin (positive vs negative)	10.759 (1.363–84.944)	0.024	0.853 (0.023–31.877)	0.932	11.363 (1.438–69.791)	0.021	3.238 (0.349–30.078)	0.878
Vimentin (positive vs negative)	11.959 (2.535–56.407)	0.002	11.213 (1.67–41.774)	0.003	12.515 (2.649–59.121)	0.001	8.386 (1.475–47.66)	0.012
TP53 (positive vs negative)	6.443 (1.815–22.873)	0.004	3.467 (0.929–12.946)	0.654	7.768 (2.161–27.918)	0.006	2.284 (0.521–10.014)	0.27

Hazard ratios with 95% confidence intervals (95% CI) were estimated and adjusted by indicated clinical parameters

## Discussion

Though both the incidence and mortality rate of cervical cancer have considerably diminished globally in the past five decades, cervical cancer remains a major cause of cancer death in women [27]. The survival of patients with primary cervical cancer is generally determined by several factors, including lymph node metastasis, parametrial invasion, tumor size, lymphovascular involvement, and histologic grade [28]. Following surgical resection of tumors, patients with one or more of the clinicopathological features mentioned above need to undergo additional therapy. Nevertheless, traditional pathological markers don’t offer reliable prognostic values to guide optimal treatment strategies. In this study, we showed that the expression of Vimentin, TP53 and Podoplanin is correlated with the survival of cervical cancer patients, indicating that the expression of these proteins may serve as valuable biomarkers to aid in the diagnosis and effective treatment of cervical cancer patients.

In this study, we employed immunohistochemistry to investigate the expression of Vimentin, TP53, and Podoplanin in cervical cancer. Our findings may help to reveal the role of these proteins in cervical cancer carcinogenesis and their prognostic significance in the management of cervical cancer patients. Vimentin, which functions as a key intermediate filament protein in mesenchymal cells, is involved in EMT and plays a critical role in the growth, invasion and metastasis of human cancer cells, including cervical cancer [3, 29, 30]. Nevertheless, it is still unknown whether or not the expression of Vimentin is correlated with the clinicopathologic features of cervical cancers. Interestingly, we found out that Vimentin protein expression is strongly associated with the onset age, lymph node metastasis, lymphatic invasion, Ki67 staining, recurrence, and survival in cervical cancer patients. Furthermore, a multivariate analysis also reveals that Vimentin expression is an independent prognostic factor for the OS and DFS of cervical cancer patients. These results indicate that Vimentin plays an important role in the malignant phenotype in cervical cancer patients. Importantly, our data suggest that positive Vimentin expression may serve as a biomarker to predict a poor prognosis in cervical cancer patients and provides important insights into the design of novel therapeutic strategies to treat cervical cancer patients.

Consistent with earlier reports [3, 31, 32], our data also showed that 20% of the cases of cervical cancer exhibit high levels of TP53 expression. Over-expression of TP53, resulting from accumulation of defected TP53 protein due to gene alterations, is commonly found in cervical cancer. Nonetheless, its prognostic value in cervical cancer remains controversial [3]. Here, we found that up-regulation of TP53 expression is correlated with the size of tumor and vascular space involvement as well as poor DFS and OS. However, when other co-variables were included in the Cox regression model, over-expression of TP53 is not an independent prognosis factor. Hence, in agreement with the findings by Åvall-Lundqvist et al. our data suggest that TP53 expression is not an independent predictive factor for patients with cervical cancer [33].

Podoplanin, selectively expressed in lymphatic endothelium, has been used to detect lymphatic invasion in several malignant neoplasms, including cervical carcinoma [34, 35]. Podoplanin, a transmembrane glycoprotein, is up-regulated in a variety of human cancer cells, especially those derived from squamous stratified epithelia (SCCs). Its expression in tumor cells is linked to increased cell migration and invasiveness [36]. It has been reported that CD44, the major hyaluronan (HA) receptor and one of the cancer stem cell (CSC) markers, is a novel partner for Podoplanin. Expression of the CD44 standard isoform (CD44s) is coordinately up-regulated together with that of Podoplanin during progression to highly aggressive SCCs in a mouse skin model of carcinogenesis, and during the process of EMT. It has been shown that aggressive squamous CSCs are enriched at the invasive front with the extracellular matrix composed of hyaluronic acids and Podoplanin [37]. Additionally, previous reports indicate that presence of lymphovascular invasion serves as a considerable risk factor for tumor recurrence in cervical squamous cell carcinoma [3]. Consistent with these reports [3, 36, 37], our data suggest that Podoplanin expression in the tumor cells displays strong association with the onset age, the existence of lymphatic invasion, lymph node metastasis, vascular space involvement, deep stromal invasion, positive parametrium, higher risk of tumor recurrence and shorter survival. Our findings also confirms that Podoplanin plays an important role in cell migration and in the lymphatic spread of cervical cancer cells to regional lymph nodes.

Ki-67 has been widely used as a proliferation marker to measure the growth fraction of human cancer cells [38]. High Ki67 expression has been suggested as a poor prognostic indicator for Ewing’s sarcomas [39] or breast cancer [40]. Although Ki-67 expression has not been found to be associated with patient’s general survival in studies conducted by us and other groups [41], we found that Vimentin expression was significantly associated with a decreased proliferation rate of cervical cancer as measured by the Ki-67 labeling index. The inversed correlation between Vimentin and Ki-67 seems to be paradoxical. However, it has been reported that cancer stem cells with increased CD44 expression tend to form the negative feedback machinery in terms of oxidative stress-induced Wnt/beta-catenin signal transduction [37, 42]. This negative feedback regulation exerted by upregulated CD44/Vimentin expression may be partially responsible for the inversed expression pattern between CD44/Vimentin and Ki-67/c-Myc [40, 41].

It is worth noting that there are some limitations regarding our research findings we presented here. First, the sample size in our study is relatively small. Thus, it is necessary to perform further studies with larger sample sizes to validate our findings. Second, a population selection bias may also exist, since it is a retrospective study. Lastly, the patients recruited in our study all had resectable tumors. Hence, it is not clear whether or not we can extend our findings to the patients with advanced nonresectable cervical cancer.

## Conclusion

As of now, clinical TNM stage doesn’t serve well as a practical indicator for the prognosis of patients with cervical cancer. Patients with the same clinical stage may display completely different clinical courses. Here, we demonstrated that Vimentin expression can act as an independent predictive factor for patients with cervical cancer, providing important guidelines for the management of cervical cancer patients.

## References

[1] Parkin DM, Bray F, Ferlay J, Pisani P (2001). Estimating the world cancer burden: globocan 2000. Int J Cancer.

[2] Ferlay J, Shin HR, Bray F, Forman D, Mathers C, Parkin DM (2010). Estimates of worldwide burden of cancer in 2008: GLOBOCAN 2008. Int J Cancer.

[3] Brabletz T, Hlubek F, Spaderna S, Schmalhofer O, Hiendlmeyer E, Jung A (2004). Invasion and metastasis in colorectal cancer: epithelial–mesenchymal transition, mesenchymal–epithelial transition, stem cells and beta-catenin. Cells Tissues Organs.

[4] Chen C, Zimmermann M, Tinhofer I, Kaufmann AM, Albers AE (2013). Epithelial-to-mesenchymal transition and cancer stem (-like) cells in head and neck squamous cell carcinoma. Cancer Lett.

[5] Zeisberg M, Neilson EG (2009). Biomarkers for epithelial–mesenchymal transitions. J Clin Investig.

[6] Yoshida GJ (2016). Emerging role of epithelial–mesenchymal transition in hepatic cancer. J Exp Clin Cancer Res.

[7] Kokkinos MI, Wafai R, Wong MK, Newgreen DF, Thompson EW, Waltham M (2007). Vimentin and epithelial–mesenchymal transition in human breast cancer–observations in vitro and in vivo. Cells Tissues Organs.

[8] Polyak K, Weinberg RA (2009). Transitions between epithelial and mesenchymal states: acquisition of malignant and stem cell traits. Nat Rev Cancer.

[9] Chikaishi Y, Uramoto H, Tanaka F (2011). The EMT status in the primary tumor does not predict postoperative recurrence or disease-free survival in lung adenocarcinoma. Anticancer Res.

[10] Perlson E, Michaelevski I, Kowalsman N, Ben-Yaakov K, Shaked M, Seger R, Eisenstein M, Fainzilber M (2006). Vimentin binding to phosphorylated Erk sterically hinders enzymatic dephosphorylation of the kinase. J Mol Biol.

[11] Thiery JP, Sleeman JP (2006). Complex networks orchestrate epithelial–mesenchymal transitions. Nat Rev Mol Cell Biol.

[12] Lin J, Liu X, Ding D (2015). Evidence for epithelial–mesenchymal transition in cancer stem-like cells derived from carcinoma cell lines of the cervix uteri. Int J Clin Exp Pathol.

[13] Chang L, Goldman RD (2004). Intermediate filaments mediate cytoskeletal crosstalk. Nat Rev Mol Cell Biol.

[14] Meek DW (2009). Tumour suppression by p53: a role for the DNA damage response?. Nat Rev Cancer.

[15] Bremer GL, Tieboschb AT, van der Putten HW, de Haan J, Arends J-W (1995). p53 tumor suppressor gene protein expression in cervical cancer: relationship to prognosis. Eur J Obstet Gynecol Reprod Biol.

[16] Chen H-Y, Hsu C-T, Lin W-C, Tsai H-D, Chang W-C (1999). Prognostic value of p53 expression in stage IB1 cervical carcinoma. Gynecol Obstet Invest.

[17] Brenna S, Zeferino L, Pinto G, Souza R, Andrade L, Vassalo J, Martinez E, Syrjänen K (2002). P53 expression as a predictor of recurrence in cervical squamous cell carcinoma. Int J Gynecol Cancer.

[18] Khunamornpong S, Siriaunkgul S, Manusirivithaya S, Settakorn J, Srisomboon J, Ponjaroen J, Thorner PS (2008). Prognostic value of p53 expression in early stage cervical carcinoma treated by surgery. Asian Pac J Cancer Prev.

[19] Bremer GL, Tieboschb AT, van der Putten HW, de Haan J, Arends J-W (1995). p53 tumor suppressor gene protein expression in cervical cancer: relationship to prognosis. Eur J Obstet Gynecol Reprod Biol.

[20] Chaffer CL, Weinberg RA (2011). A perspective on cancer cell metastasis. Science.

[21] Ordóñez NG (2006). Podoplanin: a novel diagnostic immunohistochemical marker. Adv Anat Pathol.

[22] Martín-Villar E, Megías D, Castel S, Yurrita MM, Vilaró S, Quintanilla M (2006). Podoplanin binds ERM proteins to activate RhoA and promote epithelial–mesenchymal transition. J Cell Sci.

[23] Wicki A, Lehembre F, Wick N, Hantusch B, Kerjaschki D, Christofori G (2006). Tumor invasion in the absence of epithelial–mesenchymal transition: Podoplanin-mediated remodeling of the actin cytoskeleton. Cancer Cell.

[24] Sousa B, Paredes J, Milanezi F, Lopes N, Martins D, Dufloth R, Vieira D, Albergaria A, Veronese L, Carneiro V (2010). P-cadherin, vimentin and CK14 for identification of basal-like phenotype in breast carcinomas: an immunohistochemical study. Histol Histopathol.

[25] Allegra CJ, Paik S, Colangelo LH, Parr AL, Kirsch I, Kim G, Klein P, Johnston PG, Wolmark N, Wieand HS (2003). Prognostic value of thymidylate synthase, Ki-67, and p53 in patients with Dukes’ B and C colon cancer: a National Cancer Institute-National surgical adjuvant breast and bowel project collaborative study. J Clin Oncol.

[26] Vinicius DL, Scapulatempo C, Perpetuo NM, Mohamed F, de Carvalho TS, de Oliveira ATT, Segalla JGM, Carvalho AL (2011). Prognostic and risk factors in patients with locally advanced cutaneous squamous cell carcinoma of the trunk and extremities. J Skin Cancer..

[27] Chung H, Jang M, Jung K, Won Y, Shin H, Kim J (2006). LEE HP: Cervical cancer incidence and survival in Korea: 1993–2002. Int J Gynecol Cancer.

[28] Chen C-A, Cheng W-F, Wei L-H, Su Y-N, Hsieh C-Y (2002). Radical hysterectomy alone or combined with neoadjuvant chemotherapy in the treatment of early stage bulky cervical carcinoma. J Formos Med Assoc.

[29] Luo W, Fang W, Li S, Yao K (2012). Aberrant expression of nuclear vimentin and related epithelial–mesenchymal transition markers in nasopharyngeal carcinoma. Int J Cancer.

[30] Gilles C (1996). POLETTE M, Piette J, DELVIGNE AC, Thompson EW, FOIDART JM, BIREMBAUT P: Vimentin expression in cervical carcinomas: association with invasive and migratory potential. J Pathol.

[31] Helland A, Karlsen F, Due E, Holm R, Kristensen G (1998). Mutations in the TP53 gene and protein expression of p53, MDM 2 and p21/WAF-1 in primary cervical carcinomas with no or low human papillomavirus load. Br J Cancer.

[32] Denk C, Butz K, Schneider A, Dürst M, Hoppe-Seyler F (2001). p53 mutations are rare events in recurrent cervical cancer. J Mol Med.

[33] Oh M-J, Choi J-H, Lee YH, Lee JK, Hur JY, Park YK, Lee KW, Chough SY, Saw H-S (2004). Mutant p53 protein in the serum of patients with cervical carcinoma: correlation with the level of serum epidermal growth factor receptor and prognostic significance. Cancer Lett.

[34] Kahn HJ, Marks A (2002). A new monoclonal antibody, D2-40, for detection of lymphatic invasion in primary tumors. Lab Invest.

[35] Dumoff KL, Chu C, Xu X, Pasha T, Zhang PJ, Acs G (2005). Low D2-40 immunoreactivity correlates with lymphatic invasion and nodal metastasis in early-stage squamous cell carcinoma of the uterine cervix. Mod Pathol.

[36] Martin-Villar E, Fernandez-Munoz B, Parsons M, Yurrita MM, Megias D, Perez-Gomez E, Jones GE, Quintanilla M (2010). Podoplanin associates with CD44 to promote directional cell migration. Mol Biol Cell.

[37] Yoshida GJ (2017). The heterogeneity of cancer stem-like cells at the invasive front. Cancer Cell Int.

[38] Schlüter C, Duchrow M, Wohlenberg C, Becker M, Key G, Flad H-D, Gerdes J (1993). The cell proliferation-associated antigen of antibody Ki-67: a very large, ubiquitous nuclear protein with numerous repeated elements, representing a new kind of cell cycle-maintaining proteins. J Cell Biol.

[39] López-Guerrero JA, Machado I, Scotlandi K, Noguera R, Pellín A, Navarro S, Serra M, Calabuig-Fariñas S, Picci P, Llombart-Bosch A (2011). Clinicopathological significance of cell cycle regulation markers in a large series of genetically confirmed Ewing’s sarcoma family of tumors. Int J Cancer.

[40] Yerushalmi R, Woods R, Ravdin PM, Hayes MM, Gelmon KA (2010). Ki67 in breast cancer: prognostic and predictive potential. Lancet Oncol.

[41] Davidson B, Goldberg I, Lerner-Geva L, Gotlieb WH, Ben-Baruch G, Novikov I, Kopolovic J (2000). Expression of topoisomerase II and Ki-67 in cervical carcinoma—clinicopathological study using immunohistochemistry. Apmis.

[42] Yoshida GJ, Saya H (2014). Inversed relationship between CD44 variant and c-Myc due to oxidative stress-induced canonical Wnt activation. Biochem Biophys Res Commun.

